# Combined Effects of Polydopamine-Assisted Copper Immobilization on 3D-Printed Porous Ti6Al4V Scaffold for Angiogenic and Osteogenic Bone Regeneration

**DOI:** 10.3390/cells11182824

**Published:** 2022-09-09

**Authors:** Hsi-Yao Wu, Yen-Hong Lin, Alvin Kai-Xing Lee, Ting-You Kuo, Chun-Hao Tsai, Ming-You Shie

**Affiliations:** 1 School of Dentistry, China Medical University, Taichung 406040, Taiwan; 2 X-Dimension Center for Medical Research and Translation, China Medical University Hospital, Taichung 404332, Taiwan; 3 Department of Education, China Medical University Hospital, Taichung 404332, Taiwan; 4 Graduate Institute of Biomedical Sciences, China Medical University, Taichung City 406040, Taiwan; 5 Department of Sports Medicine, College of Health Care, China Medical University, Taichung 406040, Taiwan; 6 Department of Orthopedics, China Medical University Hospital, Taichung 40447, Taiwan; 7 Department of Bioinformatics and Medical Engineering, Asia University, Taichung 41354, Taiwan

**Keywords:** additive manufacture, Ti6Al4V, dopamine, copper, angiogenesis, osteogenesis

## Abstract

Numerous studies have demonstrated that biological compounds and trace elements such as dopamine (DA) and copper ions (Cu) could be modified onto the surfaces of scaffolds using a one-step immersion process which is simple, inexpensive and, most importantly, non-cytotoxic. The development and emergence of 3D printing technologies such as selective laser melting (SLM) have also made it possible for us to fabricate bone scaffolds with precise structural designs using metallic compounds. In this study, we fabricated porous titanium scaffolds (Ti) using SLM and modified the surface of Ti with polydopamine (PDA) and Cu. There are currently no other reported studies with such a combination for osteogenic and angiogenic-related applications. Results showed that such modifications did not affect general appearances and microstructural characteristics of the porous Ti scaffolds. This one-step immersion modification allowed us to modify the surfaces of Ti with different concentrations of Cu ions, thus allowing us to fabricate individualized scaffolds for different clinical scenarios. The modification improved the hydrophilicity and surface roughness of the scaffolds, which in turn led to promote cell behaviors of Wharton’s jelly mesenchymal stem cells. Ti itself has high mechanical strength, therefore making it suitable for surgical handling and clinical applications. Furthermore, the scaffolds were able to release ions in a sustained manner which led to an upregulation of osteogenic-related proteins (bone alkaline phosphatase, bone sialoprotein and osteocalcin) and angiogenic-related proteins (vascular endothelial growth factor and angiopoietin-1). By combining additive manufacturing, Ti6Al4V scaffolds, surface modification and Cu ions, the novel hybrid 3D-printed porous scaffold could be fabricated with ease and specifically benefited future bone regeneration in the clinic.

## 1. Introduction

Commercially, titanium alloys (Ti) are extensively applied in the field of orthopedics and dentistry due to their resistance to corrosion and superior mechanical properties [1,2]. Ti has since replaced traditional materials such as Co–Cr or Ni–Cr alloys due to its toughness and excellent flexural strength [3,4,5]. The development and emergency of additive manufacturing in the field of biomedical engineering has rendered us with the capability to fabricate customizable and individualized metallic scaffolds with complex microstructures [6,7]. Amongst the various types of additive manufacturing techniques, SLM is one of the more common and suitable 3D printing approaches used for the fabrication of metallic scaffolds [8]. Even though Ti has stable chemical characteristics compared with other metallic alloys, it was reported that non-mineralized substrates still have a tendency to form on the surfaces of the scaffolds upon exposure to air, thus greatly limiting its fullest application for clinical applications. Further, a layer of dense oxide also forms on the surfaces of Ti after exposure to air, which allows for certain levels of cellular adhesion onto Ti. However, it is due to formation of such substrates that the chemical bonds between scaffolds and surrounding tissues are reduced, thus resulting in higher risks of scaffold loosening and structural weakening [9]. To solve this issue, scientists are attempting to improve bioactivity of Ti surfaces by modifying the surfaces with biological components while retaining the mechanical properties of Ti [10,11].

Surface modification is an effective method to improve bioactivity of metallic materials [12]. An increasing number of reports have been made confirming the significance of surface modification in optimizing osseointegration, osteoconductivity and osteoinductivity [13]. In recent years, the deposition and coating of dopamine (DA) onto surfaces of inert scaffolds has gained traction due to its simplicity, non-cytotoxicity and high effectiveness [14]. DA self-polymerizes into polydopamine (PDA) without the need for complex experimental setups or toxic intermediates. Our previous results have confirmed that PDA coating can significantly improve the hydrophilicity, mechanical properties and biocompatibility of a scaffold [15]. Most importantly, this PDA layer contains active functional motifs which could be used to attach other functionalities to create a diverse hybrid material. Numerous studies have further attempted to functionalize the surfaces of metallic scaffolds by adding other active metal ions, such as zinc, copper and magnesium, onto PDA-modified scaffolds which have been proven to be effective in enhancing osteogenesis [16]. As compared to traditional modification techniques such as plasma spraying and plasma immersion, PDA-assisted modifications are the current trend as it is uncomplicated, safe and inexpensive compared to traditional coating methods [17].

Trace elements such as strontium, zinc, cobalt or iron exist in our human body and have important roles to play in bone metabolism, regeneration and mineralization by acting as coenzymes or cofactors that affect downstream signaling pathways and osteogenesis cascades [18,19,20]. In addition, several trace metal ions were also reported to have angiogenic, anti-inflammatory and antibacterial capabilities [21]. Zhang et al., fabricated scaffolds with PDA and silver modifications, with results showing enhanced antibacterial effects of scaffolds. However, it was also reported that such a modification brought about a significant reduction in osteoblasts attachment [22]. Nevertheless, this study acts as a platform in inspiring us to brainstorm for other possible combinations to enhance bone tissue regenerations. In particular, the copper ion (Cu) is a trace element that can be found in the human body and is well known for its antimicrobial, osteogenic and angiogenic properties [23,24]. Recently, we modified activated carbon fibers with DA and Cu and results showed that the presence of Cu was able to increase differentiation of mesenchymal stem cells towards the osteogenic lineage [25]. According to our knowledge, there are published studies reporting on the modification of Ti with PDA and Ti with PDA/zinc ions for osteogenic applications [26]. Wang et al., fabricated a bi-functional Ti-DA-Zinc scaffolds, and results showed that such a novel scaffold had potential for bacterial inhibition and osseointegration capabilities as compared to neat Ti and Ti/PDA scaffolds [27]. Currently, there are no studies combining Ti, PDA and Cu in evaluating osteogenic and angiogenic capabilities for bone tissue regeneration; therefore, it would be interesting and novel to explore this potential for future clinical applications. 

In this study, we fabricated bifunctional Ti-PDA-Cu (TiPCu) scaffolds using SLM and step-wise immersion techniques for DA and Cu modifications. XPS and FTIR results confirmed the successful modifications of Ti with PDA and Cu ions. Furthermore, the scaffolds were able to release Cu ions in a gradual and sustained manner in accordance to the concentrations of Cu modification. Hydrophilicity, cellular adhesion, proliferation and expressions of both osteogenic and angiogenic factors were significantly improved with Cu modifications. This study could be used as a platform to motivate future studies regarding novel modifications of inert surfaces with various biological components so as to improve its potential for tissue engineering applications.

## 2. Materials and Methods

### 2.1. Fabrication of Porous Titanium Scaffolds

**Figure 1:**
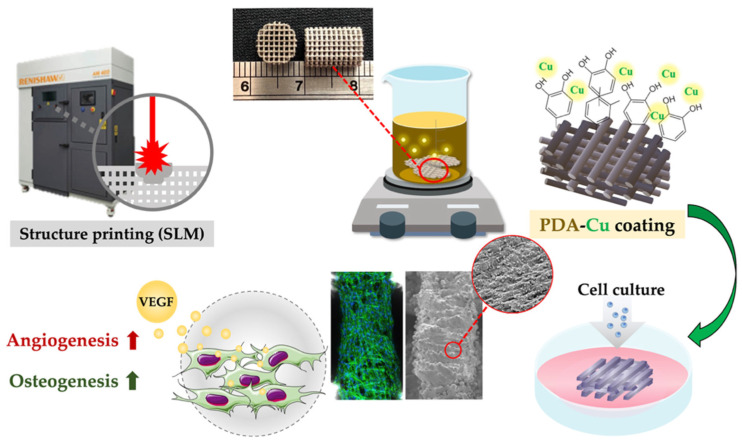
Schematic diagram of the SLM-fabricated Ti6Al4V scaffold coating with Cu ion grafted by DA, which gave the scaffold with the ability to promote angiogenesis and osteogenesis by the releasing Cu ions.

Ti6Al4V porous scaffolds were designed using commercial CAD software Solidworks and fabricated using selective laser melting (SLM; AM400, Renishaw, Wotton-under-Edge, UK) fabrication technology. Reports have shown that the pore size of the scaffold within 200–500 μm is conducive to bone ingrowth and capillary formation Therefore, in this study, each strut was designed to be 500 μm with the average diameter of the pores to be 450 μm. The fabrication parameters were as follows: average temperature of powder bed ~750 °C and chamber pressure of 5 × 10^−2^ Pa. After fabrication, the scaffolds and powder bed were left to cool down in an argon-filled chamber with a pressure of 2 × 10^4^ Pa until it reached a temperature of 100 °C and the air was then introduced into the chamber to harvest the printing specimens. The structures of the scaffolds and printing parameters were pre-determined according to our previous publications for bone scaffolds regeneration [28]. The diameter and height of the scaffolds were designed in the size of cylinder shape as 9.6 mm in height and 6.5 mm in diameter for characterization experiments and sizes of 1.6 mm in height and 6.5 mm in diameter were for cell culture assays. A schematic diagram depicting the fabrication process of our TiPCu scaffolds is shown as shown in Figure 1. The Ti scaffolds used in this study were fabricated using selective laser melting. After which, the fabricated Ti scaffolds were immersed into a polydopamine/copper (PDA/Cu) solution for surface modification via a one-step coating process that was inspired from the attachment capabilities of mussels. Finally, Wharton’s jelly mesenchymal stem cells (WJMSCs, ScienCell Research Laboratories, Carlsbad, CA, USA) and human umbilical vein endothelial cells (HUVEC, ScienCell Research Laboratories, Carlsbad, CA, USA) were evaluated for osteogenic and angiogenic capabilities. Specific details are elaborated further in their respective sections.

### 2.2. Copper Ion Coating

The scaffolds were immersed in 25 mM Tris buffer (pH 8.5) solution containing 2 mg/mL of dopamine (DA, Acros Organic, Fairlawn, NJ, USA) and together with various concentrations of CuSO_4_ (0, 25, and 50 mM; Panreac, Barcelona, Spain). The solutions were prepared and vigorously stirred for 12 h prior to immersion. After immersion, the scaffolds were placed in an ultrasonic cleaner to wash to unattached compounds and placed in an oven for the scaffolds to dry. The concentrations of DA were pre-determined according to our previous publications [6]. The following groups for experiment are denoted by the symbol Cu0, Cu25 and Cu50 as the various concentration of CuSO_4_ coating, and Ti for SLM Ti6Al4V substrate.

### 2.3. Characterization of TiPCu Scaffolds

The hydrophilicity was measured by the sessile drop method; 20 μL of deionized water was dripped onto the scaffolds and the contact angle was photographed with a microscope camera and analyzed using an ImageJ software. X-ray photoelectron spectrometer (XPS) was used to determine the chemical composition of the surfaces of the scaffolds. The scaffolds were exposed to electron incidence angles of 45° and the concentration of the measured elements (carbon (C), nitrogen (N), oxygen (O), titanium (Ti), copper (Cu)) were displayed in terms of percentages. The surface molecular conformation of the coated scaffold was determined using the Fourier transform infrared spectrometer (FTIR, Vertex 80v, Bruker, Karlsruhe, Germany) at a spectral range of 400–4000 cm^−1^ with a resolution of 2 cm^−1^. The scaffolds were sprayed with platinum, placed in a 45 °C oven to dry overnight, and then analyzed using a scanning electron microscope (SEM; JEOL JSM-7800F, Tokyo, Japan) to observe the surface morphology of the scaffolds. In addition, the element composition of the scaffold was analyzed using energy-dispersive X-ray spectroscopy (EDX). The scaffolds were compressed using a universal testing machine (EZ-test, Shimazu, Tokyo, Japan) at a loading speed of 1 mm/min. For each scaffold, the stress–strain curves, compressive strength and Young’s modulus were calculated and recorded. Ten scaffolds from each group were evaluated and the mean and standard deviation were recorded.

### 2.4. In Vitro Release Profiles of Copper

The design and size of the scaffolds are the same as that of the cell culture scaffolds that were immersed in cultured medium (#7501, ScienCell Research Laboratories, Carlsbad, CA, USA) at 37 °C. At 1, 3, 7 and 14 days, the medium collected and replaced with fresh medium. The Cu concentrations in the extracts were analyzed using inductively coupled plasma atomic emission spectrometry (ICP-AES; Perkin-Elmer OPT 1MA 3000DV, Shelton, CT, USA) and quantified the concentration of copper ions released by the scaffolds. Three independent studies were conducted and the averages were recorded.

### 2.5. Angiogenic Analysis

HUVEC used in this study were purchased and cultured with the commercial medium (#1001, ScienCell Research Laboratories, Carlsbad, CA, USA). Cells used in this study were from passages 4–6. Then, 10^6^ cells/mL of HUVEC was cultured on the Geltrex matrix (Invitrogen, Carlsbad, CA, USA) surface for 3 h and replaced the medium with scaffold extracts. Briefly, we printed scaffolds and coated them with PDA and Cu as our samples, which were subsequently washed three times with PBS, followed by disinfection in 75% ethanol at room temperature in a laminar flow for 30 min. To get the extracts of our Cu/PDA -coated scaffolds, the specimens were then immersed in the medium and placed in a 37 °C incubator with 75% humidity and 5% CO_2_ for 24 h. To assess the function of angiogenesis, we assessed the network nodes formed by the cells after 12 h. Then, the levels of vascular endothelial growth factor (VEGF, KHG0111, Invitrogen, Carlsbad, CA, USA) and angiopoietin-1 (Ang-1, EHANGPT1, Invitrogen, Carlsbad, CA, USA) were evaluated by enzyme-linked immunosorbent assay kit according to manufacturer’s instructions and with correlation to the standard curve. 

### 2.6. Cell Viability Assay and Fluorescent Staining

WJMSCs used in this study were purchased and cultured with the commercial medium (#7501, ScienCell Research Laboratories, Carlsbad, CA, USA). Cells used in this study were from passages 4–8. Prior to in vitro cell culture studies, all scaffolds were immersed in 75% alcohol and exposed to 2 h of UV light for disinfection and disinfection. The cells were seeded on the 3D-printed scaffold at a concentration of 10^6^ cells/mL and incubated in a 37 °C with humidified 5% CO_2_ atmosphere. After these cells were cultivated for a predetermined period, the cultured medium was replaced by 30 μL of PrestoBlue solution plus 270 μL of DMEM and incubated at 37 °C for 45 min in the dark. Subsequently, 100 μL of the reactant solution was transferred to new 96-well microplates. A spectrophotometer (Infinite Pro M200, Tecan, Männedorf, Switzerland) was used to read the absorbance at a wavelength of 570 nm with a reference wavelength of 600 nm. Triplicate measurements were conducted for the quantitative analysis.

For observation of cellular morphology, these 3D-printed scaffolds were rinsed with cold PBS several times and fixated using 4% paraformaldehyde (Sigma-Aldrich, St. Louis, MO, USA) for 40 min and treated with 0.1% Triton X-100 (Sigma-Aldrich, St. Louis, MO, USA) for 30 min. Fluorescent staining was performed by incubating the 3D-printed scaffolds with phalloidin conjugated to Alexa Fluor 488 (1:400, Invitrogen, Carlsbad, CA, USA) for 3 h in the dark. These samples were gently rinsed with PBS to remove excess unreacted substrates, reacted with DAPI (Invitrogen, Carlsbad, CA, USA) for 30 min in the dark and the pictures were observed using a confocal microscope (Leica TCS SP8, Wetzlar, Germany).

### 2.7. Osteogenesis-Related Protein Expressions

The WJMSCs were cultured using osteogenesis assay kits for 7 and 14 days (StemPro™ osteogenesis differentiation kit; Invitrogen, Carlsbad, CA, USA) to induce differentiation of stem cells into osteogenic cells. Then, alkaline phosphatase activity (ALP), bone sialoprotein (BSP) and osteocalcin (OC) expressions were detected at different time-points. For ALP assay, the cells were lysed in 100 μL of 1% NP40 Buffer (NP40 Cell Lysis Buffer), then a pNPP alkaline phosphatase assay kit (BioAssay Systems, Biocore, NSW, Australia) was utilized for determination of ALP levels. The total protein content was measured using a BCA protein detection kit (Thermo Scientific, Waltham, MA, USA), and the relative ALP activity was calculated as the change in absorbance divided by the total protein content. For the BSP and OC measurements, the protein levels were evaluated using an ELISA kit (Invitrogen, Carlsbad, CA, USA) following the manufacturer’s instructions. The protein concentrations were calculated based on a correlation with a standard curve.

### 2.8. Statistical Analysis

A one-way statistical analysis of variance (ANOVA) was applied to analyze the significance of the difference between the different experimental groups in each experiment. Determination of the significant deviation of each sample was carried out using Scheffe’s multiple comparison test. A *p* value < 0.05 was considered statistically significant, as indicated by “*” or “#” in the different group comparisons.

## 3. Results and Discussion

### 3.1. Characterization of TiPCu Scaffolds: Morphology and Contact Angle 

**Figure 2:**
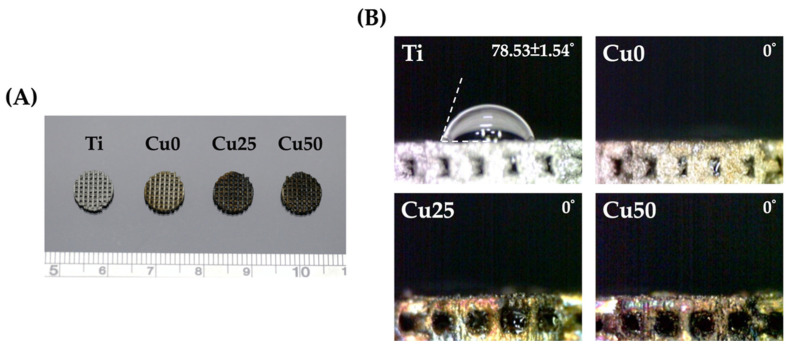
(**A**) The appearance of printed structure before and after PDA/Cu coating, with the increase of Cu concentration, the appearance of the scaffold becomes darker. (**B**) Contact angle of water droplets on the scaffold. Except for the untreated group, the other three groups exhibited a super hydrophilic state. Data presented as mean ± SEM, *n* = 6 for each group.

Figure 2 shows photo images of the fabricated scaffolds and the scaffolds were designed to be circular in shape with a diameter of 1 cm. Surface modification with PDA and Cu did not affect the designs of the scaffolds except for color differences due to the presence of Cu. The surfaces of the scaffolds were covered with a network of intact and interconnected pores with uniform porosity. Interconnected pores are important for efficient hard tissue regeneration. Native bone structures exhibit a gradient of porosity ranging from dense cancellous bones to cortical bones. Even though there were conflicting results regarding the ideal porosities of scaffolds, it is generally recommended that pore sizes of approximately 200 µm to 400 µm are beneficial for new bone tissue ingrowth into scaffolds [29,30]. In addition, there were reports stating that porosity of scaffolds should be approximately 50 to 90% or otherwise equivalent to trabecular bones so as to allow efficient diffusion of nutrients. Therefore, our Ti scaffolds were fabricated according to the reported publications of others and also with references to our previous publications [31]. 

Surface hydrophilicity is also considered as a primary requirement for the prediction of subsequent cellular behaviors and activities. Various studies have confirmed that hydrophilicity has a role to play in determining the subsequent potential of a scaffold to stimulate cellular adhesion, proliferation and bone-scaffold integration [31]. Figure 2B shows that hydrophilicity improved with the addition of PDA. As seen, the addition of PDA made Ti scaffolds so hydrophilic that it interfered with the surface tension of the water droplet, thus disrupting the spherical shape of the droplet. The main reason for such an observation is due to the fact that PDA is highly hydrophilic as it contains a high amount of catechol moieties. Generally, a water contact angle of less than 75° is known to be beneficial for subsequent cellular behavior [32]. There are currently conflicting results regarding super-hydrophilicity of surfaces, with reports mentioning that contact angle of less than 5° were unconducive for cellular adhesion and attachment due to poor adsorption of proteins on super-hydrophilic surfaces. However, it is still important to note that adhesion and attachment of cells are not solely regulated by hydrophilicity alone, and thus further tests are required to confirm for the efficacy of our scaffold modifications for tissue engineering applications.

### 3.2. Characterization of TiPCu Scaffolds: Chemical Composition, Bioactivity and Mechanical Test

**Figure 3:**
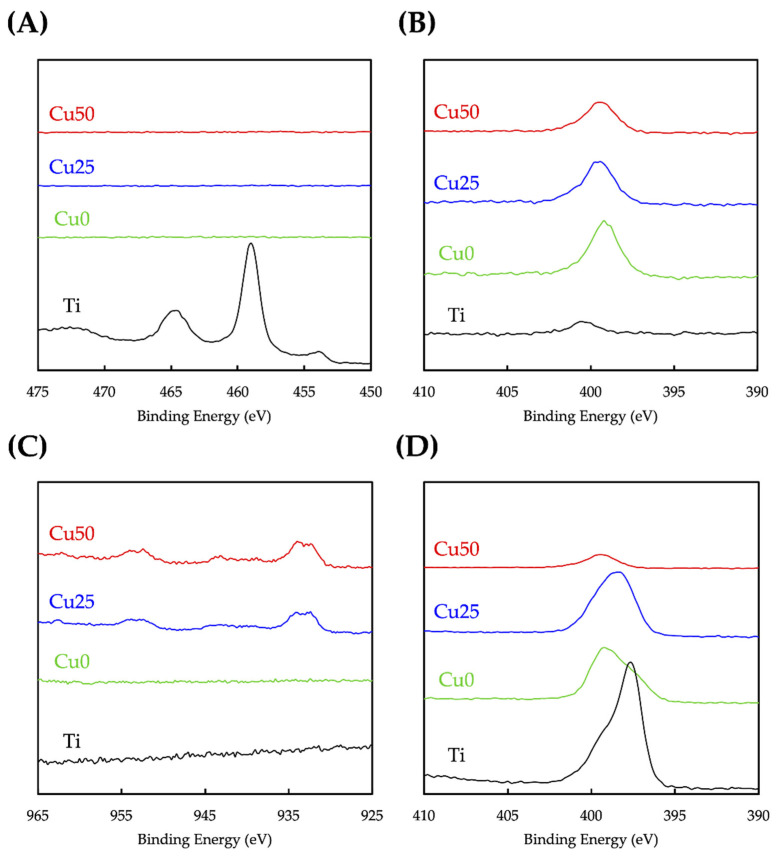
XPS spectra of (**A**) Ti, (**B**) N, (**C**) Cu, (**D**) O of the 3D-printed Ti6Al4V scaffolds before and after PDA/Cu coating.

**Figure 4:**
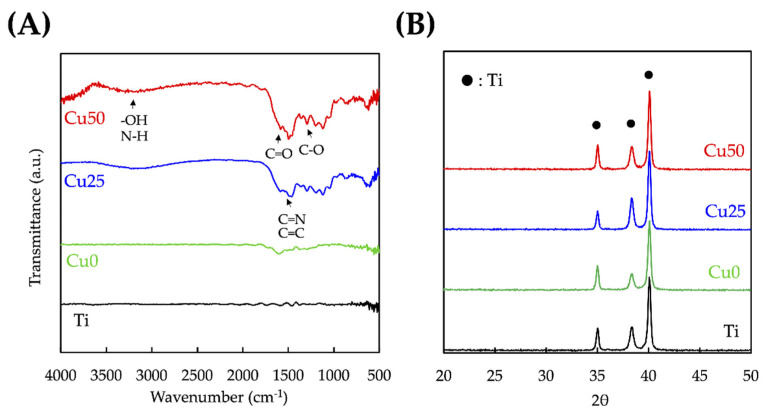
(**A**) FTIR and (**B**) XRD profiles of the 3D-printed Ti6Al4V scaffolds before and after PDA/Cu coating.

The surface modifications of Ti with PDA and Cu were confirmed using XPS analysis and as shown in Figure 3. As seen from the results, the high-resolution spectra of Ti and O confirmed that the entire surface of the scaffolds were coated with PDA and/or Cu due to the absence of Ti spectra from Cu0, Cu25 and Cu50. Figure 3B shows that PDA coating was successful as seen from the presence of N spectra. The reduction in N spectra, coupled with the presence of Cu spectra in both Cu25 and Cu50, confirmed the successful loading of varying concentrations of Cu onto the scaffolds [33]. Taken together, it could be seen that the surfaces of Ti scaffold could be easily modified using the one-step immersion process. The existence and comparison of the different functional groups between Cu0, Cu25 and Cu50 were analyzed using FTIR and are shown in Figure 4A. Cu was noted to be functionalized and attached to PDA via the following bonds: -OH, N–H at 3250 cm^−1^, C=O at 1640 cm^−1^, C=N, C=C at 1600 cm^−1^ and C–O at 1400 cm^−1^ [34]. The increase in peak intensity of the above bonds is due to increase in Cu and PDA functionalization. The crystalline structures of the scaffolds were detected using XRD and as shown in Figure 4B. There were multiple Ti inherent characteristic peaks at the 2θ values of 35, 38.5 and 40. The above results further confirmed that PDA and Cu were successfully modified onto Ti via covalent bonds and that the modification did not affect the presence of Ti [31]. Furthermore, it was possible to modify the surfaces of Ti with varying concentrations of substrates, thus allowing us to fabricate scaffolds with individualized functions.

**Figure 5:**
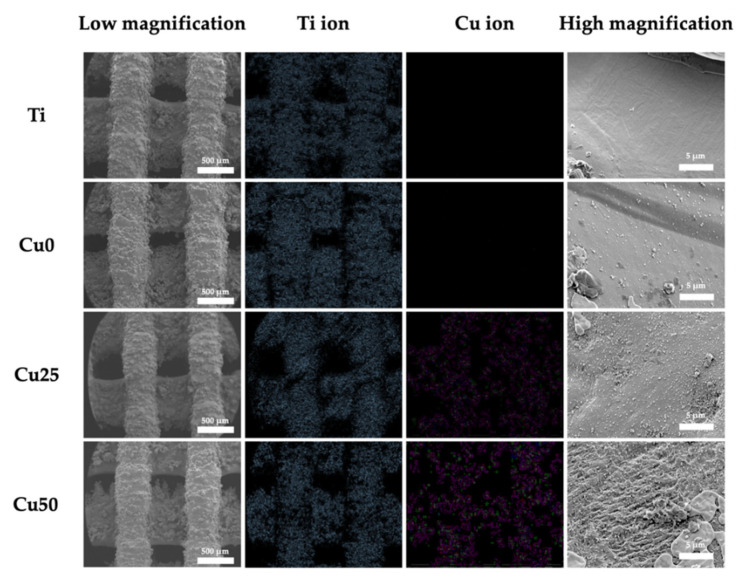
Low-magnification and high-magnification SEM microscopic images of the 3D-printed Ti6Al4V scaffolds before and after PDA/Cu coating, and their elemental mapping image of Ti and Cu ions.

SEM/EDX images were used to compare the surface morphology of the various scaffolds. As seen in Figure 5, the pores were well interconnected and the struts were able to support the weight of the scaffold. As mentioned, pores are important for tissue ingrowth, diffusion of nutrition and act as anchor points for cell adhesion. Upon higher magnification, it could be seen that the surfaces of Ti were relatively smooth, while there was a gradual increment of surface roughness from Cu0 and Cu25 to Cu50. There were micro-clusters of PDA deposition on the surfaces of Cu0, while rough grooves and contours were noted on the Cu50 scaffolds [35]. The rough surfaces caused by PDA and Cu deposition were hypothesized to be beneficial for subsequent cellular behaviors as increased surface roughness was reported to enhance entrapment of fibrin proteins, thus leading to improved adhesion of osteogenic cells and scaffold stability. There were numerous other scientific reports stating on the positive correlation between surface roughness, cellular attachment, proliferation and osteogenic capabilities [36,37,38].

**Figure 6:**
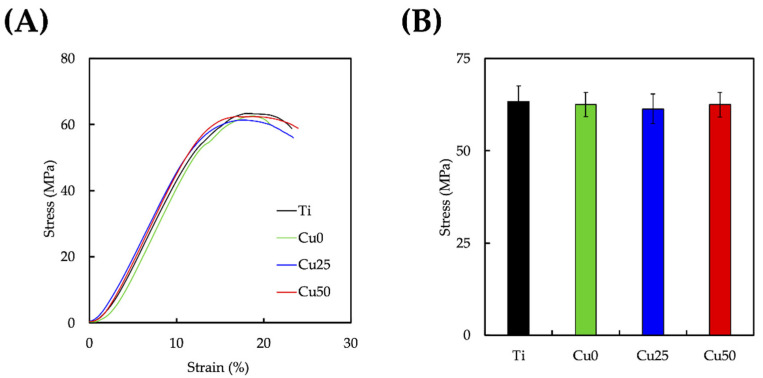
(**A**) The stress–strain profile and (**B**) the breaking point of various 3D-printed Ti6Al4V scaffolds. Data presented as mean ± SEM, *n* = 6 for each group.

The strain–stress curves showed that the TiPCu scaffolds were first elastically stretched, followed by deformation, and ultimately leading to fracture of the scaffold [39]. As noticed in Figure 6, beginning from the point of compressive strength, the stress on the scaffold is almost at its elastic stage and is stepped up linearly with distance since the scaffold deformation does not change, while the pressure exerted by the probe of the device is transmitted directly to the specimen platform through the scaffold. The TiPCu scaffold had the greatest breaking point averaging of 63.4 ± 4.2 MPa and compressive modulus of 472.2 ± 12.5 MPa in the strain range of 0.5–11.5%, without significant differences in all testing groups. Due to the increased toughness of Ti itself, it produces a large deformation to resist external force when subjected to axial pressure, while the coating procedure did not seem to affect the overall mechanical properties of the scaffold.

### 3.3. In Vitro Release Profiles of Copper

**Figure 7:**
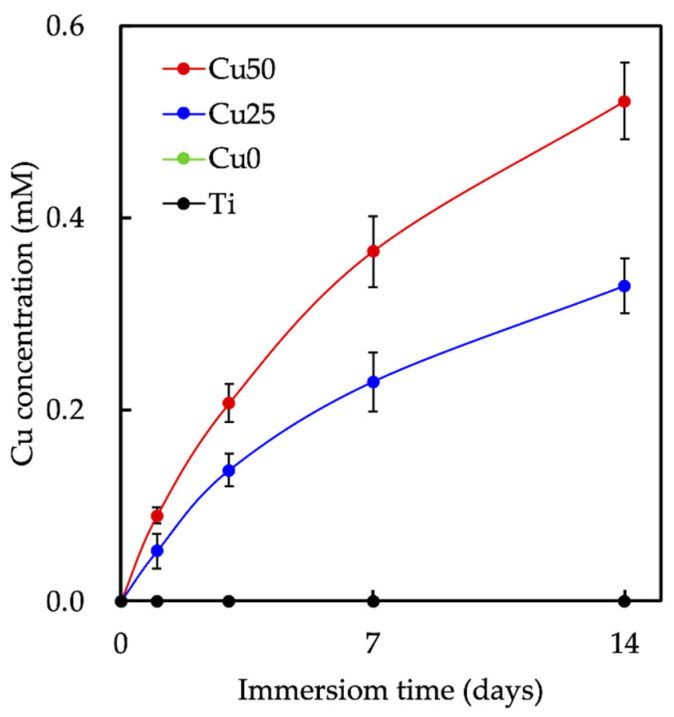
The concentration of Cu released from the scaffolds immersed in medium. Data presented as mean ± SEM, *n* = 6 for each group.

Cu release profiles for Cu25 and Cu50 were evaluated, as illustrated in Figure 7. For both groups, a sharp increase in Cu release was noted during the first 3 days of immersion before gradually declining. After 14 days of immersion, both Cu scaffolds were still shown to release Cu ions in a stable manner. This is beneficial for both osteogenesis and angiogenesis as both regenerative processes might require a few weeks for complete regeneration depending on the size of the defect. In addition, Cu50 was found to have 1.5× higher levels of Cu after 14 days of immersion. Cu has been shown to increase proliferation of endothelial cells and in vivo angiogenesis. In the clinical settings, physicians have attempted to reduce angiogenesis of tumors by restricting Cu intake [40]. Recently, Wu et al., incorporated Cu into ceramic scaffolds and showed that the release Cu ions induced hypoxia inducible factor-1α secretion from MSC, thus promoting expressions of various angiogenic-related proteins [41]. A recent study by Kong et al., showed that Cu had a positive impact on angiogenesis via increasing angiogenic-related growth factor secretions from fibroblast and endothelial cells. Cu is also commonly used in the area of bone regeneration due to its antibacterial and collagen stimulation capabilities [42]. 

### 3.4. In Vitro Angiogenesis Effect of Cu/PDA Coating

**Figure 8:**
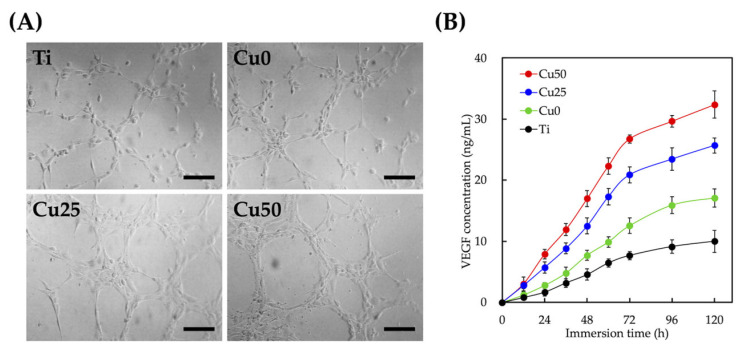
(**A**) Tube formation assay of HUVEC with different extracts treatment. Scale bar = 100 μm. (**B**) VEGF secretion from HUVEC cultured with different extract for 5 days. Data are presented as mean ± SEM, *n* = 6 for each group.

The effect of Cu/PDA coating on the angiogenesis of HUVECs was studied by the tube formation analysis. Tube formation is a traditional method for considering endothelial cells differentiation behaviors in vitro. As for the tube formation assay, the HUVEC treated with Cu25 and Cu50 advancingly assembled more tubes compared with Ti and Cu0 (Figure 8A). In our previous study, Cu ion was shown to be pro-angiogenic via upregulating VEGF production [43]. Therefore, the secretion of VEGF was evaluated as shown in Figure 8B. The time-dependent secretion kinetics demonstrated that all the scaffolds demonstrated similar secretion trends, with rapid secretion for the first 48 h with equilibrium at 72 h. However, the various scaffolds differ in their final VEGF secretion rate, with Ti, Cu0, Cu25 and Cu50 reaching a final secretion capacities of approximately 8%, 15%, 30% and 42%, respectively. Previous studies indicated that VEGF play an important factor in the development and formation of the angiogenic process [44]. The stimulatory effect of Cu on angiogenesis is due to the upregulation of VEGF expression and the KDR activation after those cells treated with Cu-contained extracts [42]. Therefore, the above results indicated that our scaffolds were able to induce VEGF secretion in a stable manner, thus also indicating that Cu-modified Ti scaffolds have the capability to enhance angiogenesis in a dose-dependent manner. Cu/PDA-coated Ti scaffolds used in this study were demonstrated helpful for the first time for tube formation of HUVEC. 

**Figure 9:**
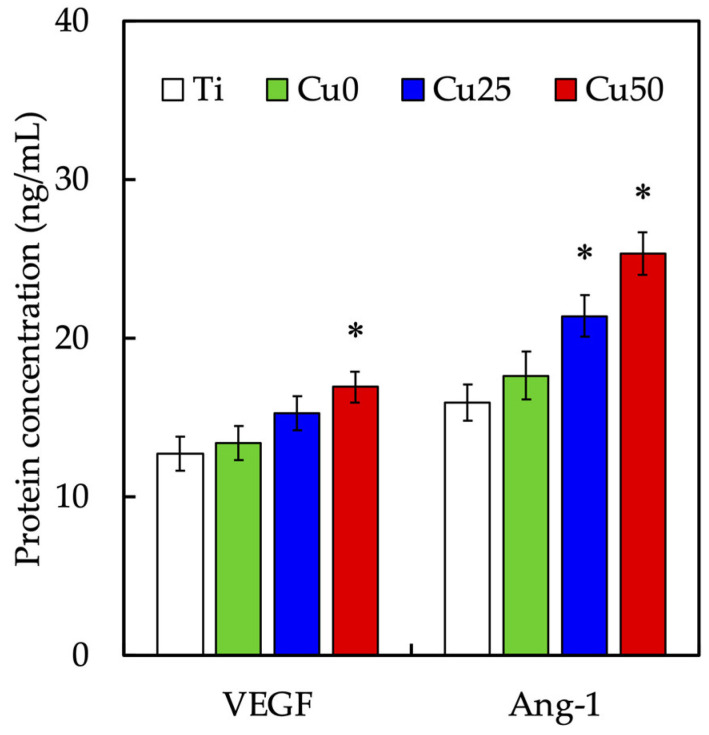
Angiogenic-related differentiation marker of VEGF activity and Ang-1 expression of HUVEC cultured on different TiPCu scaffolds at 3 days. * indicates a significant difference (*p* < 0.05) from the Ti group. Data are presented as mean ± SEM, *n* = 6 for each group.

The levels of VEGF and Ang-1 secreted from WJMSCs were evaluated using an ELISA kit and shown in Figure 9. After 3 days of culture, it could be seen that Cu50 had significantly higher levels of both VEGF and Ang-1 secretion as compared to Ti. There was approximately an increase of 33.0% VEGF and 59.2% Ang-1 from the Cu50 scaffolds. On the other hand, there was no significant difference between VEGF secretion of Cu25 and Ti. However, Cu25 was noted to have a 1.34-fold increase of Ang-1 as compared to Ti. Various kinds of angiogenic-related biomarkers are required at different stages for blood vessel development. Initially, the recruitment, proliferation and differentiation of the endothelial cells require high concentrations of VEGF for tube formation [39]. Subsequently, the maturation and stabilization of blood vessels require other growth factors, such as Ang-1, to weaken after the cell proliferated. VEGF is a family of growth factors that work via tyrosine kinase receptors on endothelial cells and are reported to be induced during hypoxic conditions. Of all the VEGF, VEGF-A is the most studied and is known to bring about migration of endothelial cells, create fenestration and subsequent creation of vascular lumen. Sen et al., indicated that Cu utilized similar pathways used by hypoxia to induce VEGF secretions [45]. It was shown that Cu concentrations as low as 0.005 mM was sufficient to induce angiogenesis [46]. In the present study, the effective concentrations of Cu ions in Cu25 ranged from 0.05 mM to 0.33 mM and in Cu50 ranged from 0.09 mM to 0.52 mM. The toxicity levels of Cu on MSCs were reported be around the range of 0.25 mM; however, our studies reported on cumulative Cu concentrations over a period of 14 days, which were shown to have no cytotoxicity on cells and yet were able to promote vascular tube formation [47].

### 3.5. Cell Viability Assay and Fluorescent Staining

**Figure 10:**
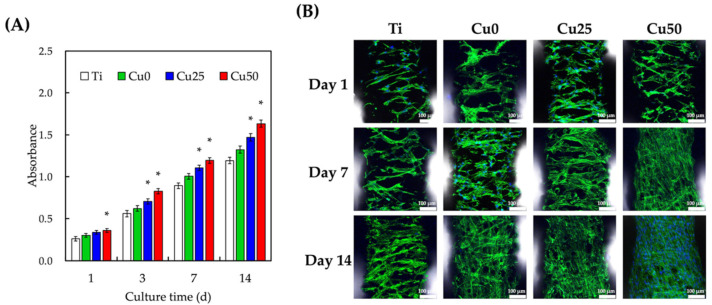
(**A**) Cell proliferation and (**B**) F-actin (green) and DAPI (blue) staining of WJMSCs on the TiPCu 3D scaffolds at various time points. * indicates a significant difference (*p* < 0.05) from the Ti group. Data are presented as mean ± SEM, *n* = 6 for each group. The scale bar is 100 µm.

The quantification results of WJMSCs proliferation and morphology cultured on the various scaffolds were as shown in Figure 10. All scaffolds provided WJMSCs proliferation after 1 day of culture and proliferation increased exponentially with regards to the durations of culture (Figure 10A). However, it is interesting to note that Cu50 had significantly higher levels of proliferation as compared to Ti after just 1 day of culture. After day 14 of culture, Cu had approximately 1.4-times higher levels of proliferation as compared to Ti alone. On the other hand, cellular proliferation between Cu0 and Ti was not significant, while Cu25 had significantly higher levels of proliferation from day 3 onwards. The morphological results from Figure 10B showed that WJMSCs were better adhered onto the surfaces of scaffolds as compared to Ti and Cu0. In comparison, cells in the Cu50 groups were spindle-like and well spread as compared to cells in Ti. These findings hinted that the presence of Cu enhanced cellular proliferation. It was further hypothesized that the increased surface roughness and hydrophilicity from Cu contributed to the increase in early cellular proliferation by improving initial cellular adhesion [48]. Furthermore, there were published studies reporting that Cu ions were able to influence and regulate Wnt signaling and downstream β-catenin and PI3K/Akt activation, thus increasing cellular resistance to apoptosis [49,50].

### 3.6. Osteogenic-Related Protein Expression

**Figure 11:**
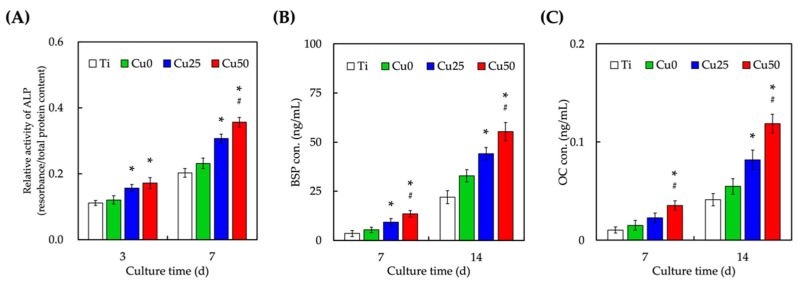
Osteogenic-related differentiation marker of (**A**) ALP, (**B**) BSP and (**C**) OC expression of WJMSCs cultured on different TiPCu scaffolds at 7 and 14 days. * indicates a significant difference (*p* < 0.05) from the Cu0 group. # indicates a significant difference (*p* < 0.05) from the Cu25 group. Data are presented as mean ± SEM, *n* = 6 for each group.

The levels of osteogenic-related markers ALP (Figure 11A), BSP (Figure 11B) and OC (Figure 11C) were evaluated after 7 and 14 days of culture. The presence of Cu enhanced secretion of all osteogenic-related markers at all time points in a dose-dependent manner. Cu25 and Cu50 had significantly higher levels of ALP, BSP and OC as compared to Ti after 7 and 14 days of culture. In fact, Cu50 was also found to have significantly higher levels of ALP, BSP and OC as compared to Cu0 at all time points. As compared to Ti, our statistical results also showed that levels of all osteogenic-related markers were significantly enhanced after 3 and 7 days of culture with Cu50. ALP is an early maker of osteogenic differentiation and has a huge role to play in regulation of early bone mineralization. ALP works by hydrolyzing inorganic pyrophosphate and promoting the formation of hydroxyapatite, thus activating the processes of mineralization. On the other hand, BSP is part and parcel of the extracellular matrix of bones and is secreted by osteoblasts, odontoblasts and osteoclasts. Therefore, as compared to ALP, BSP is commonly used as a late-stage mineralization marker. Lastly, OC is a late-stage bone matrix protein commonly used as a marker for bone turnover regardless of normal physiological or pathological conditions. Several studies had been demonstrated that osteogenic differentiation and vessel formation in bone tissue engineering were closely coupled [51]. In fact, the ultimate goal of bone regeneration is the building of sufficient quality and quantity of the vascularized bone with function [52]. It was indicated Cu ions induced osteogenesis, which enhanced new bone regeneration in vivo [53]. Therefore, several studies demonstrated Cu ions induced osteogenic differentiation that also promoted new bone regeneration in vivo [54]. Taken together, it could be seen that Cu was able to enhance both in vitro early and late osteogenic markers, thus having the potential for in vivo osteochondral regeneration. Ti is a common material used for medical and dental applications and devices due to its mechanical, biological and anticorrosive characteristics [55]. In this study, we have shown that Ti could be further modified via a one-step immersion process which could bring about enhanced cellular adhesion, proliferation and subsequent angiogenesis and osteogenesis capabilities. This study again showed that the concept of osseointegration is critical in designing a good biological scaffold for clinical applications. Even though more studies are required to confirm for the efficacy of the modifications, we are confident that this study brings us a step closer to the surface modification of biomaterials for tissue engineering related clinical applications and studies.

## 4. Conclusions

The SLM fabricated porous Ti scaffolds were successfully modified with PDA and Cu ions as proven by the FTIR and XRD results. In addition, the incorporation of PDA and Cu did not affect the microstructural characteristics and porosity of Ti. Hydrophilicity was significantly enhanced with the incorporation of Cu. Immersion results showed that the scaffolds were able to release Cu ions in a stable and gradual manner for 14 days of immersion. Time-dependent adsorption kinetics of VEGF showed that all scaffolds had similar uptake trends with adsorption equilibrium at 72 h. However, Cu50 had the highest adsorption capacity of 42%. Cellular activities and behaviors such as adhesion, proliferation and differentiation were positively affected by the Cu modifications. Lastly, angiogenic-related markers, such as Ang-1 and VEGF, and osteogenic-related markers, such as ALP, BSP and OC, were significantly enhanced with Cu modifications in a dose-dependent manner. We believe that such one-step immersion modification could be used as a simple methodology to include different organic and biological compounds on surfaces of 3D-printed Ti6Al4V scaffolds so as to enhance the potential for use of these biomaterials in bone tissue regeneration in the clinic.

## Data Availability

Data are available in a publicly accessible repository.
